# 非EB病毒病原体所致感染相关噬血细胞综合征的临床特征及预后

**DOI:** 10.3760/cma.j.issn.0253-2727.2022.02.007

**Published:** 2022-02

**Authors:** 亚红 尤, 晶石 王, 昭 王

**Affiliations:** 首都医科大学附属北京友谊医院 Department of Hematology, Beijing Friendship Hospital, Capital Medical University, Beijing 100050, China

**Keywords:** 噬血细胞综合征, 感染, 临床特征, 预后, Hemophagocytic lymphohistiocytosis, Infection, Clinical characteristics, Outcome

## Abstract

**目的:**

探讨非EB病毒病原体所致感染相关噬血细胞综合征（IAHLH）患者的临床特征及预后。

**方法:**

收集2015年1月至2021年3月首都医科大学附属北京友谊医院确诊的48例非EB病毒病原体所致IAHLH患者的临床资料，对患者的临床特征、治疗、疗效及预后进行回顾性分析。

**结果:**

共纳入48例患者，男28例，女20例，中位年龄34.5（2.0～74.0）岁，导致IAHLH的病原体中，病毒16例（33.3％），细菌17例（35.4％），寄生虫13例（27.1％），真菌2例（4.2％），患者发病至确诊噬血细胞综合征（HLH）的中位时间为40（10～160）d，发病至确诊为IAHLH的中位时间为67（23～270）d。患者起病时临床表现如下：发热48例（100％），脾大34例（70.8％），血细胞减低38例（79.1％），铁蛋白升高45例（93.8％），甘油三酯升高7例（14.6％），纤维蛋白原降低17例（35.4％），NK细胞活性减低26例（59.1％），可溶性CD25升高35例（74.5％）。25例（52.1％）患者起病时伴淋巴结肿大。明确导致HLH的病原体后尽快减停细胞毒药物及激素并应用有效的抗感染治疗，36例（75.0％）患者获得完全缓解，93.3％（14/15）的寄生虫及真菌所致IAHLH患者获得了病情缓解，细菌及病毒相关IAHLH仅有66.7％（22/33）的缓解率。患者5年预期总生存（OS）率为72.3％（95％ *CI* 50.3％～69.8％），多因素分析显示，总胆红素大于2倍正常上限（*OR*＝20.0，95％ *CI* 1.1～378.3，*P*＝0.046）及诱发HLH的病原体感染未控制（*OR*＝19.9，95％ *CI* 2.9～134.5，*P*＝0.002）为预后不良因素。

**结论:**

非EB病毒病原体所致IAHLH预后较好，诊断后应尽快减停细胞毒药物及激素，有效控制病原体感染为关键性治疗。

## Abstract (en)

**Objective:**

To explore the clinical characteristics and outcomes of patients with non-Epstein-Barr virus (EBV) infection-associated hemophagocytic lymphohistiocytosis (IAHLH).

**Methods:**

Clinical data of 48 patients diagnosed with non-EBV IAHLH in Beijing Friendship Hospital from January 2015 to March 2021 were collected, and the clinical characteristics, treatment, curative effect and prognosis of the patients were analyzed retrospectively.

**Results:**

This study included 48 patients, 28 males and 20 females, with a median（range）age of 34.5（2–74）years. Pathogens that cause IAHLH were as follows: virus（16 cases, 33.3％）, bacteria（17 cases, 35.4％）, parasitic agents（13 cases, 27.1％）, and fungi（2 cases, 4.2％）. The median time from onset to diagnosis of hemophagocytic syndrome（HLH）was 40（10–160）days. The median（range）time duration from prodrome to the definite diagnosis of IAHLH was 67（23–270）days. The clinical characteristics were fever（48 cases, 100％）, splenomegaly（34 cases, 70.8％）, cytopenia（38 cases, 79.1％）, elevated ferritin（45 cases, 93.8％）, elevated fasting triglyceride levels（7 cases, 14.6％）, hypofibrinogenemia（17 cases, 35.4％）, decrease natural killer cell activity（26 in 44 cases, 59.1％）, and elevated sCD25（35 cases, 74.5％）. Twenty-five patients（52.1％）had adenopathy. Once a certain pathogen was identified as the causative factor of hemophagocytic lymphohistiocytosis（HLH）, cytotoxic agents and glucocorticoids were withdrawn, and specific pathogen-directed treatment was initiated. After treatment, 36 cases（75.0％）achieved complete response, and 14 of 15 patients（93.3％）with parasitic and fungal HLH got a response; however, the response rate of patient with bacterial and viral HLH was only 66.7％（22 of 33 patients）. The estimated 5-year overall survival rate was 72.3％（95％ *CI* 50.3％–69.8％）. The adverse prognostic factors were total bilirubin over the upper limit of normal（*OR*＝20.0, 95％ *CI* 1.1–378.3, *P*＝0.046）and pathogenic infection not fully controlled（*OR*＝19.9, 95％ *CI* 2.9–134.5, *P*＝0.002）.

**Conclusion:**

Non-EBV IAHLH has a good prognosis. When diagnosed, cytotoxic agents and glucocorticoids should be tapered off, and pathogen-targeted therapy should be critically administered to clear the triggering infection.

噬血细胞性淋巴组织细胞增多症（hemophagocytic lymphohistiocytosis, HLH）又称噬血细胞综合征，是一组由先天基因缺陷或后天继发性因素导致的危及生命的炎症因子风暴，HLH常见的继发性因素为感染、恶性肿瘤、自身免疫性疾病及其他（如移植、妊娠等特殊免疫抑制状态）[1]–[3]。感染相关HLH（IAHLH）是欧美国家继发性HLH最常见的形式，病原体包括病毒、细菌、真菌、寄生虫等，我国多中心数据显示IAHLH发病率在所有HLH患者中位居第二，仅次于肿瘤相关HLH[3]–[4]。EB病毒（EBV）是亚洲国家IAHLH最常见的病因，EBV-HLH常表现为复发难治性HLH，异基因造血干细胞移植有助于改善患者的结局，但EBV-HLH患者中位总生存（OS）时间仅3.5个月，OS率仅20％左右[5]–[7]。值得注意的是，非EBV（non-EBV）病原体导致的IAHLH在临床上较常见，且患者经治疗后预后较好，但病原体及临床特征的多样性为non-EBV IAHLH的诊断和治疗带来了巨大挑战，目前多为个案报道，国内缺乏相关研究。因此，本研究对近年来在本中心确诊的non-EBV IAHLH患者进行回顾性分析，探讨其临床特征及预后。

## 病例与方法

1. 病例：回顾性分析2015年1月至2021年3月在首都医科大学附属北京友谊医院确诊的non-EBV IAHLH病例。

2. 诊断标准：①符合HLH-2004诊断标准[8]；②未检测到HLH相关缺陷基因突变；③排除自身免疫性疾病、肿瘤、药物等其他病因所致HLH；④外周血EBV-DNA及组织中EBV编码的小RNA（EBER）均为阴性；⑤通过血液或组织DNA检测、血清学检测、质谱分析等方法明确是病原体感染且临床多学科判定该病原体是诱发HLH的唯一致病因素。

3. 治疗：患者诊断HLH后积极筛查HLH病因，注意感染性疾病的鉴别诊断，积极寻找感染病原体证据并进行经验性治疗。尽量避免在诊断HLH后立即应用细胞毒药物及激素。如患者HLH病情活动，且病因筛查暂无结论，可考虑应用依托泊苷联合激素控制病情。对于难治复发的HLH患者，应用DEP方案（脂质体阿霉素+依托泊苷+甲泼尼龙）控制病情[9]。一旦明确non-EBV IAHLH，立即针对病原体进行治疗，不再继续应用细胞毒药物，并同时减停激素。

4. IAHLH疾病状态评估：评价指标包括临床症状及实验室检查。临床症状包括发热及病原体感染症状是否得到有效控制。实验室检查包括血常规、肝功能、血清铁蛋白、纤维蛋白原、甘油三酯、组织噬血现象、NK细胞活性、可溶性CD25（sCD25）水平。缓解定义为临床症状缓解，同时上述实验室检查项目中至少两项改善≥25％[10]。

5. 随访：随访方式为查阅患者住院、门诊病例及电话随访，随访截止时间为2021年5月1日。OS时间定义为自发病至随访截止日期或因各种原因死亡的时间。

6. 统计学处理：采用SPSS 23.0软件进行统计学分析。患者的人口学和临床特征采用描述性统计学分析。所有非正态分布的计量资料采用中位数（范围）表示。分类变量采用*χ*^2^检验进行组间比较。采用Kaplan-Meier曲线进行生存分析，Log-rank检验进行组间比较。将单因素分析时*P*<0.1的变量纳入Cox回归进行多因素分析。*P*<0.05定义为差异具有统计学意义。

## 结果

1. 病原体类型及确诊方法：共收集48例non-EBV IAHLH患者的临床资料，病原体类型及确诊方法见表1。病毒、细菌、寄生虫、真菌等病原体类型均可导致IAHLH。对于non-EBV IAHLH，病毒中最常见的病原体为巨细胞病毒及人类免疫缺陷病毒；细菌中最常见病原体为分枝杆菌中的结核分枝杆菌，其次为布氏杆菌，非结核分枝杆菌感染也会导致IAHLH，如鸟分枝杆菌、麻风分枝杆菌；寄生虫IAHLH中内脏利什曼病相关HLH最常见。此外，真菌中的曲霉菌、组织胞浆菌感染均可能导致HLH。

**表1:** 48例非EB病毒所致感染相关噬血细胞综合征患者的病原体及确诊方法

病原体	例数（％）	确诊方法
病毒	16（33.3）	
巨细胞病毒	11（22.9）	PCR（血液、肺泡灌洗液）
人类免疫缺陷病毒	2（4.2）	血清学+确证试验（HIV-1/HIV-2抗体）
人类疱疹病毒6型	1（2.1）	PCR（血液）
汉坦病毒	1（2.1）	血清学（IgM/IgG）
戊型肝炎病毒	1（2.1）	血清学（IgM/IgG）+PCR
细菌	17（35.4）	
结核分枝杆菌	7（14.6）	痰液/组织抗酸染色+PCR
布氏杆菌	5（10.4）	血培养
李斯特菌	1（2.1）	血、脑脊液培养
肺炎支原体	2（4.2）	血清学（IgM）
麻风分枝杆菌	1（2.1）	组织病理+质谱分析
鸟分枝杆菌	1（2.1）	组织PCR
寄生虫	13（27.1）	
利什曼原虫	11（22.9）	骨髓涂片+PCR
肺吸虫	1（2.1）	血清学（IgM）
疟原虫	1（2.1）	血涂片+PCR
真菌	2（4.2）	
曲霉菌	1（2.1）	肺泡灌洗液培养
组织胞浆菌	1（2.1）	骨髓涂片+质谱分析

2. 临床及实验室特征：患者中位发病年龄为35（2～74）岁，男28例（58.3％），无明显性别差异。3例患者为小于14岁儿童，其余均为成人患者。患者起病至确诊HLH的中位时间为40（10～160）d，起病至明确致病病原体并诊断IAHLH的中位时间为67（23～270）d，较诊断HLH约晚3周。34例（70.8％）患者初诊时有脾脏肿大，不同病原体感染导致脾大的分布情况为：细菌9例（56.3％），病毒12例（70.6％），寄生虫12例（92.3％），真菌1例（50.0％）。此外，半数以上的患者有局部或系统性淋巴结肿大，不同类型病原体致IAHLH患者出现淋巴结肿大的分布情况为：细菌7例（43.8％）、病毒11例（64.7％），寄生虫6例（46.2％），真菌1例（50.0％）。其中8例患者进行了淋巴结活检并送病理，6例患者病理显示为淋巴组织反应性增生，其余2例患者通过组织基因分子学检查发现致病病原体（分别为巨细胞病毒和鸟分枝杆菌）。发病前，约75％患者既往身体健康，有6例患者有慢性基础疾病且发病时原有基础疾病均处于稳定状态，此外有1例结核相关HLH患者发病时为妊娠状态。

对于non-EBV IAHLH，所有患者在起病时均有发热症状，约90％的患者发现铁蛋白升高、骨髓等组织中有噬血现象，约80％患者出现至少两系血细胞减低，在诊治过程中，半数患者需要输注红细胞或血小板改善临床症状。本研究中，仅有35.4％的患者血浆纤维蛋白原降低至1.5 g/L以下。在生化实验室检查方面，三分之一以上的患者出现乳酸脱氢酶升高，以寄生虫感染患者尤为明显。另外，甘油三酯水平升高超过3.0 mmol/L的患者仅占14.6％。约半数患者出现肝功能异常，主要表现为合成功能减弱（白蛋白降低，66.7％），肝细胞破坏（转氨酶升高，52.1％），胆道酶升高（碱性磷酸酶、γ-谷氨酰转肽酶升高，20％～40％），代谢异常（总胆红素升高，18.8％）。HLH特异性检查提示，NK细胞活性减低及sCD25升高可见于半数以上患者。不同类型病原体致IAHLH患者的临床特征见表2。

**表2:** 不同类型病原体致感染相关噬血细胞综合征患者的临床特征［例（％）］

特征	病毒	细菌	寄生虫	真菌	总计
淋巴结肿大	7（43.8）	11（64.7）	6（46.2）	1（50.0）	25（52.1）
LDH>2ULN	7（43.8）	4（25.0）	7（53.8）	0（0）	18（37.5）
HLH指标					
发热	16（100.0）	17（100.0）	13（100.0）	2（100.0）	48（100.0）
脾大	9（56.3）	12（70.6）	12（92.3）	1（50.0）	34（70.8）
血细胞减低	16（100.0）	12（70.6）	10（76.9）	1（50.0）	38（79.1）
SF>500 µg/ml	16（100.0）	16（94.1）	13（100.0）	1（50.0）	45（93.8）
TG>3.0 mmol/L	2（12.5）	1（5.9）	3（23.1）	1（50.0）	7（14.6）
FBG<1.5 g/L	9（56.3）	10（58.8）	10（76.9）	2（100.0）	17（35.4）
NK细胞活性减低^a^	10（71.4）	8（50.0）	7（58.3）	1（50.0）	26（59.1）
骨髓噬血现象	14（87.5）	16（94.1）	12（92.3）	2（100.0）	43（89.6）
sCD25升高^b^	12（75）	10（71.4）	10（83.3）	2（100.0）	34（77.3）
肝功能异常					
转氨酶>2ULN	10（62.5）	6（37.5）	8（61.5）	1（50.0）	25（52.1）
TBIL>2ULN	5（31.3）	1（6.3）	2（15.4）	1（50.0）	9（18.8）
白蛋白<30 g/L	11（68.8）	11（64.7）	10（76.9）	0	32（66.7）
ALP>2ULN	3（18.8）	3（17.6）	3（23.1）	1（50.0）	10（20.8）
γ-GGT>2ULN	9（56.3）	7（41.2）	5（38.5）	1（50.0）	22（45.8）
输血	11（68.8）	7（41.2）	6（46.2）	1（50.0）	25（52.1）
β_2_-MG升高^c^	9（81.8）	9（69.2）	8（80.0）	1（50.0）	27（75.0）

注：ULN：正常值上限；LDH：乳酸脱氢酶；HLH：噬血细胞综合征；SF：血清铁蛋白；TG：甘油三酯；FBG：纤维蛋白原；NK细胞：自然杀伤细胞；sCD25：可溶性CD25；TBIL：总胆红素；ALP：碱性磷酸酶；γ-GGT：γ-谷氨酰转肽酶；β_2_-MG：β_2_-微球蛋白；^a^ 病毒、细菌、寄生虫、真菌组检测总例数分别为14、16、12、2例；^b^ 病毒、细菌、寄生虫、真菌组检测总例数分别为16、14、12、2例；^c^ 病毒、细菌、寄生虫、真菌组检测总例数分别为11、13、10、2例

3. 治疗及疗效：几乎所有患者在诊断HLH时病因并未明确，因此non-EBV IAHLH的诊断往往滞后，尽量避免诊断HLH后立即应用细胞毒药物及激素。48例患者中，5例患者临床表现为难治/复发HLH，甚至需要应用DEP方案进行挽救治疗，但重新评估患者并筛查病因后诊断为IAHLH。non-EBV IAHLH的治疗主要包括三方面：首先是病因治疗，积极控制病原体感染是治疗的关键，也是防止复发的重要举措，病原体感染的诊治依据热带病研究所及感染科等的综合意见。其次是对症支持治疗，对于血细胞减低的患者，应用粒细胞集落刺激因子（G-CSF）提升WBC，对症输注血液制品改善贫血、减轻出血风险非常有必要。此外，要警惕过于积极地应用细胞毒药物及大剂量激素进行诱导缓解治疗，且一旦non-EBV IAHLH诊断明确，尽量避免继续应用细胞毒药物并应减停激素，静脉输注人免疫球蛋白可起到协同抗感染作用。此外，芦可替尼可用于控制患者发热等细胞因子风暴表现。患者的治疗方案及疗效见表3。

**表3:** 48例非EB病毒所致感染相关噬血细胞综合征患者的治疗方案及疗效

病原体	治疗方案		缓解例数/总例数（％）
	确诊病原体前	确诊病原体后	
病毒			10/16（62.5）
巨细胞病毒	激素+依托泊苷/芦可替尼	更昔洛韦、膦甲酸钠	8/11（72.7）
人类免疫缺陷病毒	芦可替尼	抗逆转录病毒	1/2（50.0）
人类疱疹病毒6型	激素	更昔洛韦	0/1（0）
汉坦病毒	丙种球蛋白+激素	利巴韦林	1/1（100.0）
戊型肝炎病毒	丙种球蛋白+激素	利巴韦林	0/1（0）
细菌			12/17（70.6）
结核分枝杆菌	丙种球蛋白+激素/DEP方案	联合抗结核治疗	4/7（57.1）
布氏杆菌	激素/依托泊苷+芦可替尼	利福平+多西环素	4/5（80.0）
李斯特菌	激素/依托泊苷/DEP方案	美罗培南	0/1（0）
肺炎支原体	丙种球蛋白+激素	阿奇霉素	2/2（100.0）
麻风分枝杆菌	激素	氨苯砜+利福平+氯苯酚	1/1（100.0）
鸟分枝杆菌	依托泊苷+激素/DEP方案	阿奇霉素+阿米卡星+氯法齐明	1/1（100.0）
寄生虫			12/13（92.3）
利什曼原虫	激素+依托泊苷/DEP方案	葡萄糖酸锑钠/两性霉素B	10/11（90.9）
肺吸虫	−	吡喹酮	1/1（100.0）
疟原虫	丙种球蛋白+激素	青蒿素	1/1（100.0）
真菌			2/2（100.0）
曲霉菌	依托泊苷	两性霉素B	1/1（100.0）
组织胞浆菌	激素+依托泊苷/DEP方案	两性霉素B	1/1（100.0）

注：DEP方案：脂质体阿霉素+依托泊苷+甲泼尼龙；−：未应用控制噬血细胞综合征的药物治疗

经治疗，36例（75％）患者HLH获得缓解。约90％以上寄生虫及真菌致IAHLH患者病情获得了缓解。病毒及细菌致IAHLH患者治疗后缓解率较低，但仍有65％左右的患者获得了缓解。

4. 预后：患者中位随访26（1～80）个月，至随访截止，所有已经获得缓解的患者未出现HLH复发。5年预期OS率为72.3％（95％*CI* 50.3％～69.8％）。以病原体感染是否得到控制进行分组，结果显示，原发感染控制组的患者5年OS率为85.9％（95％ *CI* 62.0％～78.2％），明显高于感染未控制组（5年OS率为0）（*P*<0.001）。所有患者中死亡12例，其中7例为诱发HLH的病原体感染未得到控制（2例为巨细胞病毒感染，1例为李斯特菌感染，1例为人类免疫缺陷病毒感染，其余3例为结核分枝杆菌感染）。另外5例患者中2例患者病原体（布氏杆菌、利什曼原虫）感染已得到有效控制，但HLH未缓解，患者最终死亡；其余3例患者分别因粒细胞缺乏期重症感染、戊型肝炎病毒感染继发肝脏衰竭、放弃治疗而死亡。

为进一步分析各项实验室指标及治疗因素对生存的影响，对初诊时患者WBC、HGB、PLT、是否累及中枢神经系统、肝功能、铁蛋白、纤维蛋白原、sCD25、原发感染是否控制等进行单因素分析，结果显示PLT<20×10^9^/L、总胆红素高于2倍正常上限、纤维蛋白原<1.5 g/L、sCD25>30 000 pg/ml、累及中枢神经系统、病原体感染未控制是影响患者OS的不良因素（*P*值均<0.05）。但是多因素分析显示，影响患者OS的不良预后因素为：总胆红素高于2倍正常上限（*OR*＝20.0，95％*CI* 1.1～378.3，*P*＝0.046）及诱发HLH的病原体感染未控制（*OR* ＝19.9，95％*CI* 2.9～134.5，*P*＝0.002）。纤维蛋白原减低（*OR*＝0.08，95％ *CI* 0.01～1.08，*P*＝0.058）对预后的影响不显著。

## 讨论

在一种或多种病因作用下系统性免疫功能紊乱并产生细胞因子风暴为HLH的特征。HLH可表现为多器官、多系统受累，患者出现发热、脾大、血细胞减低、肝功能异常，同时表现为细胞毒性T细胞及巨噬细胞系统异常，如sCD25升高、NK细胞活性减低、铁蛋白升高、噬血现象等[1],[11]。2 197例成人HLH患者的统计数据显示，IAHLH在所有成人HLH中占50.4％，其中70.2％为non-EBV病原体所致[3]。日本的一项研究发现，non-EBV病原体是小于1岁及大于15岁IAHLH患者最常见的诱因（占50％以上），而EBV是1～15岁IAHLH患者的最常见诱因[12]。我国多中心数据显示，2005至 2014年诊断的601例患者中，IAHLH患者197例，non-EBV IAHLH患者70例，仅占IAHLH患者的35.5％ [4]。而本中心近7年诊断non-EBV IAHLH共48例，且发现多种既往鲜有报道的病原体类型，如李斯特菌、麻风分枝杆菌、鸟分枝杆菌等。国内外统计数据均显示病毒是最常见的病原体类型[3]–[4]。但在本研究中，病毒及细菌所致IAHLH比例相当，寄生虫中的利什曼原虫致HLH患者的比例较既往明显升高。更值得一提的是，本研究也是目前样本量最大的系统性临床研究，本中心数据显示，随着HLH尤其是IAHLH诊断技术的提升，我们对此疾病的诊断效率提升，同时也显示了多学科诊治的关键性。

与既往文献报道一致，本研究未发现性别差异，自出现HLH首发症状至诊断HLH的间隔时间较文献报道延长1～2周[13]–[14]，仔细筛查病因后诊断IAHLH的时间又较HLH滞后约3周。文献报道IAHLH患者49％～58％出现脾大[15]–[16]。但本研究中，高达70.8％的患者初诊时影像学检查有脾大证据，病毒相关HLH更为常见。半数以上患者出现淋巴结肿大，其中病毒IAHLH最为常见，肿大淋巴结取活检并送病理及病原体检测有助于排查肿瘤性病变，同时进一步明确感染病原体。文献报道，结核分枝杆菌所致IAHLH多数（50％～85％）发生于免疫功能受抑制或伴慢性疾病患者[15],[17]，本研究中仅29％的结核分枝杆菌所致HLH有伴随疾病或处于妊娠状态。此外，本研究75％以上患者起病时无伴随疾病，这一差异可能与患者分布及就诊科室不同相关。

所有患者均在发病后出现反复发热症状，同时约80％患者出现至少两系血细胞减低，且半数以上需要输注血制品改善临床症状，这一表现与骨髓衰竭性疾病类似，因此需要进一步检查以明确诊断。与文献报道相似，约半数患者出现肝功能异常，其中以白蛋白降低及转氨酶升高为主要表现，此外，仅14.6％的non-EBV IAHLH患者甘油三酯水平超过3.0 mmol/L，远低于HLH患者70％的发生率[18]。

目前，国内外尚无公认的non-EBV IAHLH治疗方案。本研究发现治疗致病病原体感染最为关键，所有HLH达到缓解的患者原发感染均得到控制。既往文献对于是否使用免疫制剂尚有争论，但病因诊断的滞后性不容忽视，同时考虑到HLH病情的严重性，细胞毒药物及激素的使用不可避免，但需非常慎重。值得一提的是，5例难治/复发HLH患者在重新筛查病因后找到病原体，所以及时发现并诊断non-EBV IAHLH是治疗的关键。经治疗，75％患者获得缓解且无HLH复发，缓解率显著高于EBV-HLH患者[6],[9]。芦可替尼作为JAK通路抑制剂，已被报道用于改善HLH患者的临床症状和减轻系统性炎症反应，以控制体温、改善病情[19]。本研究中应用芦可替尼的患者例数较少，需要更多的数据。

我国单中心数据显示EBV-HLH患者1年的死亡率为78％，异基因造血干细胞移植后的OS率仅为52.8％[6]。另外，经典的HLH-04方案治疗成人HLH患者的1年OS率仅35％[20]。本研究发现non-EBV IAHLH患者的5年OS率约为72.3％，其预后显著优于EBV-HLH。本研究的IAHLH患者中，约三分之一的患者起病时出现纤维蛋白原降低，低于HLH患者[18]。纤维蛋白原为肝脏合成的一种急性时相反应蛋白，参与凝血过程，同时调控免疫细胞的抗微生物功能，以达到限制病原体扩散的作用[21]。Flick等[22]报道，纤维蛋白原是白细胞整合素Mac-1的生理性配体，是调控炎症反应的重要成分。在新型冠状病毒肺炎（COVID-19）患者中，纤维蛋白原升高提示持续感染，同时也是抵抗病毒感染的保护因素[21]。本研究中，纤维蛋白原降低是预后的不良因素，但多因素分析显示其预后意义不显著。既往报道显示，胆红素升高为HLH患者的不良预后因素，我们也得到同样的结论[20],[23]。本研究证实，影响HLH预后的因素主要是原发感染的控制情况及起病时总胆红素水平。

本研究系统总结了non-EBV IAHLH患者的临床特征并进行了预后分析，为此类患者的诊治提供了可靠的临床依据，及早明确诊断并进行有效的抗感染治疗是最关键的举措。
